# Macrophage activation syndrome in a newborn infant born to a untreated mother with adult onset still disease

**DOI:** 10.1186/1546-0096-12-S1-P216

**Published:** 2014-09-17

**Authors:** Jung Woo Rhim, Soo Young Lee, Joo Hyung Park, Soon Joo Lee, So Young Kim, Dae-Chul Jeong

**Affiliations:** 1 Pediatrics, College of Medicine, The Catholic University of Korea, Daejeon, Korea, Republic of; 2 Pediatrics, College pf Medicine, The Catholic University of Korea, Incheon, Korea, Republic of; 3 Pediatrics, College of Medicine, The Catholic University of Korea, Seoul, Korea, Republic of

## Introduction

In neonatal lupus, macrophage activation syndrome (MAS) is very rare. Now, we reported a newborn infant with MAS born from a untreated mother with adult onset Still disease.

## Objectives

In infant born from a mother with autoimmune disease, clinical manifestations of neonatal MAS were similar with neonatal sepsis. We have to consider neonatal MAS from a mother with autoimmune disease.

## Methods

We reported a newborn infant with macrophage activation syndrome (MAS) born from a mother with positive anti-nuclear (ANA) and anti-SSA/Ro antibodies. The 2,500 g girl was born at 37^+6^ weeks of gestation in good condition. Mother had been diagnosed with adult onset Still disease 10 years previously. During pregnancy, she did not take any medication as she was free of symptoms. The baby was admitted due to tachypnea and fever 12 hours after birth. Initial laboratory findings showed mild anemia with thrombocytopenia, and mild elevation of alanine aminotransferase (ALT). Anti-platelet antibody was not detected. A work-up of infectious etiology, including agents responsible for congenital infection, was negative.

## Results

On the 10^th^ hospital day (HD), the baby showed severe abdominal distension caused by hepatosplenomegaly, and persistent, high fever despite empirical antibiotic therapy. We identified positive ANA and anti-SSA/Ro antibodies from the infant, compatible with those found in the mother. The baby's electrocardiography was normal. On the 18^th^ HD, she showed deterioration of overall condition with high ferritin, ALT, and profound thrombocytopenia. The baby received intravenous immunoglobulin, steroid (pulse and oral), and cyclosporine. Gene study for perforin, K-ras, and N-ras was negative. Her general condition showed improvement after treatment, although mild fever and organomegaly remained. We maintained high dose steroid and cyclosporine, and all medication was tapered and stopped at 12 weeks of age.

## Conclusion

We suggest that transplacental transfer of maternal auto-antibodies may be associated with the infant's MAS.

## Disclosure of interest

None declared.

